# New Canary Islands Roman mediated settlement hypothesis deduced from coalescence ages of curated maternal indigenous lineages

**DOI:** 10.1038/s41598-024-61731-x

**Published:** 2024-05-15

**Authors:** Vicente M. Cabrera

**Affiliations:** Department of Biochemistry, Microbiology, Cell Biology and Genetics, Universidad de La Laguna, 38200 San Cristobal de La Laguna, Spain

**Keywords:** Evolution, Genetics

## Abstract

Numerous genetic studies have contributed to reconstructing the human history of the Canary Islands population. The recent use of new ancient DNA targeted enrichment and next-generation sequencing techniques on new Canary Islands samples have greatly improved these molecular results. However, the bulk of the available data is still provided by the classic mitochondrial DNA phylogenetic and phylogeographic studies carried out on the indigenous, historical, and extant human populations of the Canary Islands. In the present study, making use of all the accumulated mitochondrial information, the existence of DNA contamination and archaeological sample misidentification in those samples is evidenced. Following a thorough review of these cases, the new phylogeographic analysis revealed the existence of a heterogeneous indigenous Canarian population, asymmetrically distributed across the various islands, which most likely descended from a unique mainland settlement. These new results and new proposed coalescent ages are compatible with a Roman-mediated arrival driven by the exploitation of the purple dye manufacture in the Canary Islands.

## Introduction

The Canary Archipelago is located approximately 108 kms off Morocco’s southwestern Atlantic coast. It is made up of seven oceanic islands geographically and administratively divided into two provinces. The eastern province includes three of the islands named Gran Canaria, Lanzarote and Fuerteventura, and the western province the four remaining islands of Tenerife, La Gomera, La Palma and El Hierro. The eastern islands are geologically older and, due to their proximity to the Sahara desert, drier than the western ones but also more accessible by sea. Since the European maritime expansion along the Atlantic Africa in the fourteenth century, the Canary Islands attracted special attention as the only Archipelago of the area inhabited by indigenous people with a late Neolithic culture. The numerous and multidisciplinary studies carried out on this population have recently been reviewed from archaeological^1^ and genetic perspectives^2^. New radiocarbon dates based on short-life samples, allowed the construction of a robust chronological model for the islands hypothesizing a permanent settlement on the Archipelago around the turn of the epoch^3^. On the other hand, new sequencing methodologies have revolutionized the analysis of ancient DNA improving success for sequencing mitogenomes and whole genomes from archaeological specimens^4^. Applying these techniques to indigenous remains from the Canary Islands, a northern African origin of their most recent ancestors has been redefined^5–7^. However, the bulk of the data from the indigenous remains of the Canary Islands have been obtained with Polymerase Chain Reaction (PCR) techniques and subsequent classic Sanger sequencing^6,8–10^. Regrettably, these techniques are prone to contamination and sequencing artefacts^11^. The potential existence of such disturbing phenomena were suspected when the ancient haplotypes were contrasted with the largest (n = 896) sample studied so far of extant whole mtDNA Canarian genomes^12^. In addition, a disparity exists between archaeological and genetic ages, with the latter being much older^13^. Perhaps, the biggest failure of studies about the indigenous settlement of the Canary Islands is the absence of a model capable of integrating the data gathered from the different scientific disciplines in a coherent framework.

The aims of the present study are: (a) To perform a critical re-analysis of the published mtDNA indigenous haplotypes in order to clear up those contaminant types that obscured correct results; (b) To apply updated mtDNA evolutionary rates^14,15^ towards obtaining more realistic coalescent ages for the indigenous lineages; (c) To reformulate the timing and sources of the Roman-mediated indigenous settlement in order to incorporate the archaeological and genetic data into a congruent narrative.

## Results

### Contamination and sequencing artefacts in the Canary Islands indigenous maternal genetic pool

Studies of ancient DNA (aDNA) based on the Polymerase Chain Reaction (PCR) and subsequent Sanger sequencing have been shown to provide unreliable data^11^. This possibility increases when new haplotype were detected for the first time in aDNA studies. This was the case for the previously-recognized indigenous mtDNA genetic pool of the Canary Islands. From the 81 different lineages found in the published studies to date, 15 (19%) were not reported in the historical or current populations from the Canary Islands or in any continental regions where the most likely ancestors originated (Table S1). Table 1, lists ten (12%) of the haplotypes that might have resulted from partial sample contamination or incomplete sequencing.

**Table 1:** Possibly contaminated and/or incompleted Canarian native haplotypes, based on HVS1 variants (16,000 to 16,400 range) minus 16,000.

Detected	Hg1	Most probable	Contaminator	Hg2	Region
093 192	H/HV/U/R	093 192 (256 270 362)	Incomplete	U5a1b4	IP (68)
129 294	H/HV/U/R	(126) 129 294 (296 304)	Incomplete	T2b	IP (75)
145 213	H/HV/U/R	145 213 (223 278 294 390)	Incomplete	L2	NWA (11)
126 255 292 294	T2c1d3	126 292 294	069 126 255	T2c1d3	NWA (11)
239 278	U*	(172 219) 239 278	Incomplete	U6a1a1	NWA (30)
172 219 221 224 278	U6a	172 219 278	221 224 311	U6a7	NWA (6)
129 169 172 189 213	U6c	129 169 172 189	213	U6c1	NWA (42)
126 223 262 320	L3e2b	223 320	126 262 292 294	L3e2	NA (70)
223 239 278 292	L3	(111A 145 184) 223 239 278 292 (311 355 390)	Incomplete	L2e	Senegal (6)
223 278 311 355 362	L3b1a12	(114A 129 213) 223 278 311 355 362	Incomplete	L2b1a	Mali (71)

Hg1 and Hg2 mean haplogroup classification before and after the analysis.

The fact that the first hypervariable segment (HVSI) studied was amplified in seven overlapping small fragments^8^, favored the formation of these chimeric lineages. In order to evaluate the authenticity of the results, samples were also tested for haplogroup diagnostic positions by restriction fragment length polymorphisms (RFLPs) for phylogenetic consistency between HVSI sequences and RFLPs^8^. However, in the case of ambiguous HVSI haplotypes, a contaminated haplogroup RFLP assay could paradoxically misclassify that haplotype. A case in question could be the pair of transitions 16,172–16,278 that by RFLP (7028 Alu−; 3010 Tsp+) was classified as belonging to haplogroup H1, but has also been found as belonging to haplogroup U6a in Morocco^16^, on the Portuguese island of Madeira, very close to the Canary Islands^17^, and within the U6a7a1a Acadian clade of French origin^18^. In addition, at least the 16,213 transition was a likely sequence artefact^11^ as it was found in different haplotypes of independent haplogroups in which it was not previously detected (Table S1).

### Persistency and phylogeographic origin of the Canary Islands indigenous haplotypes

**Figure 1:**
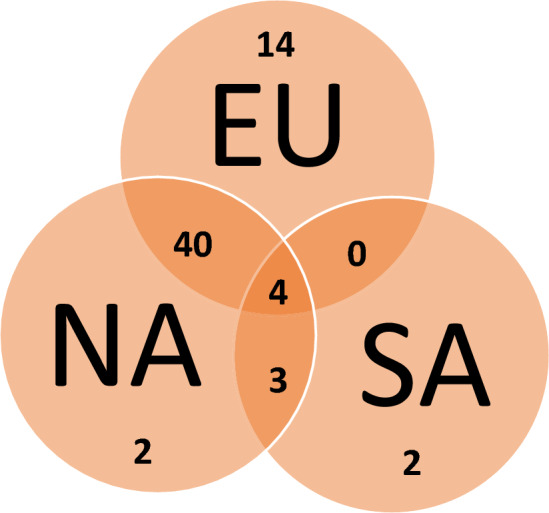
Venn diagram showing the indigenous haplotype overlapping among Europe (EU), North Africa (NA) and sub-Saharan Africa (SA).

It was recently found^12^ that around 50–60% of the Canary Islands indigenous mtDNA lineages are extant in the current Canary Island populations. However, when all the lineages detected in the historic and present-day samples were re-evaluated a slow decreasing trend was discerned, since the historic times indigenous lineages represented 37.9% of the total, whereas in present-day samples they account only for 26.5% of all the lineages observed (Table S1). Because all the published results from various disciplines point to a Canarian indigenous’ North African origin, the abundance of exclusive matches of indigenous haplotypes to Europeans compared with North Africans was surprising in our results (*p* = 0.006). A graphical representation, including sub-Saharan Africa populations, showed that most indigenous haplotypes matched to both North Africans and Europeans, while those from sub-Saharan African were in the minority (Fig. 1).

**Figure 2:**
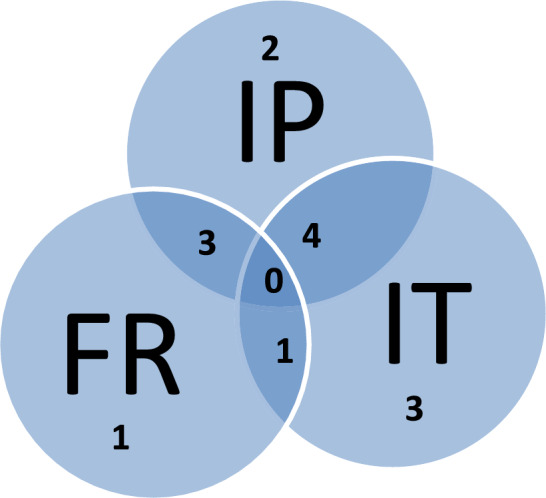
Venn diagram showing the indigenous haplotype overlapping among Iberian Peninsula (IP), France (FR) and Italy (IT).

About half of the indigenous haplotypes detected in El Hierro matched to Europeans alone. A partition of the 14 indigenous haplotypes shared exclusively with Europe (Fig. 2) suggested that the contribution of the Iberian and Italian peninsulas pair might be greater (20.6%) than that of Iberia and France (11.5%), but was not statistically significant (*p* = 0.17).

The relative affinities of the indigenous Canarian to the northern African regions (Table S1), suggested that 67.3% of the matches occurred to both northwest and northern Africa. However, exclusive matches with the northwest (26.5%) were significantly higher (p = 0.01) than to the northern region (6.1%). Of the 81 indigenous haplotypes examined, 33 (41%) were not detected in historic or contemporary samples from the Canary Islands (Table S1). Of these, 7 haplotypes (9%) exclusively matched to European regions (marked with an asterisk in Table 3 and with two asterisks in Table S1). These haplotypes could have not yet been detected in northern Africa, but it is also possible that they were brought to the islands by European males and, since mtDNA is transmitted by females, they went extinct on the Canary Islands. Similarly, there are also exclusive matches of indigenous haplotypes with the Middle East and sub-Saharan Africa that lack a congruent explanation (Table S1). There are haplotypes detected solely in the Canary Islands and South America, most probably due to the post-conquest forced migration of Canarian natives to that continent (Table S1). Another interesting case are those haplotypes derived from the autochthonous haplogroup U6b1a with prominent implantation in western islands^6^ that although lacking exact matches, still have their closest counterparts in the Moroccan sister clade U6b1b^19^. Putatively sub-Saharan African haplotypes of the haplogroup L3b1a12 detected in the eastern island of Gran Canaria^5,6,20^, whose HVSI region (16223-16278-16311-16362), exact matches were within haplogroup L3b1a11 from Madagascar^21^. However, the complete sequencing of several L3b1a12 indigenous mtDNA genomes^5,6,20^ revealed that the Canarian haplotypes differ from their putative African counterparts by six unique transitions in their coding region (8697, 9947, 10646, 11257, 14136, 14553), differing from the phylogenetic identity inferred from the HVSI analysis. Taken together, this denotes the need to study complete mitogenomes to obtain reliable genetic matches.

### Divergence of indigenous genetic pool among islands

**Figure 3:**
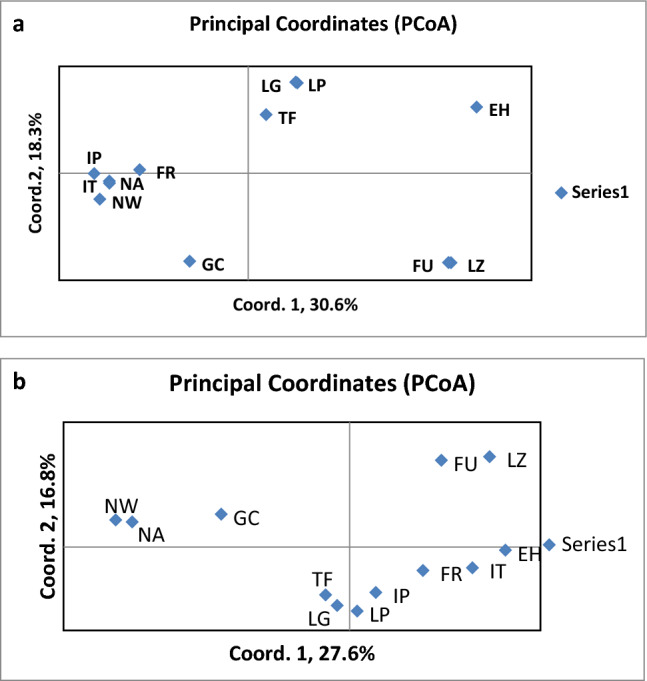
Principal Coordinate Analyses showing the relative affinities among islands (LZ, Lanzarote; FU, Fuerteventura; GC, Gran Canaria; TF, Tenerife; LG, La Gomera; LP, La Palma and EH, El Hierro) and with the Continental African (NW, North West Africa; NA, Northern Africa) and European regions (IP, Iberian Peninsula; FR, France; IT, Italy) from where their putative ancestors came. (**a**) Distances between continental regions based on their relative sharing of indigenous haplotypes. (**b**) Distances between continental regions based on their own haplotype pools.

Present-day Canary Islands insular populations’ suggest that genetic differentiation between the western (Tenerife, La Gomera, La Palma, and El Hierro) and eastern (Gran Canaria, Lanzarote, and Fuerteventura) islands may date back to pre-colonial times^22^, corroborated by recent mitogenome and whole genome analyses of the indigenous populations^5–7^. A pair-wise match-distance between islands is in Table S3, and a graphical representation of their respective relationships and genetic affinities with their putative continental colonizers is in Fig. 3.

In principal coordinates’ analysis (Fig. 3a), the genetic match distances between continental regions are based on their respective sharing of Canarian indigenous haplotypes exclusively. The coordinate 1 axis clearly separates all the continental regions samples from those of the Canary Islands, meaning that they primarily share the same ancestral haplotypes. Coordinate 2 axis, in turn, separated the western from the eastern Canary Islands, with the least sampled eastern islands of Fuerteventura and Lanzarote and the westernmost island of El Hierro, showing the greatest genetic drift effects^23^. On the other hand, Gran Canaria was the island that shared most indigenous lineages with their putative continental maternal sources. In Fig. 3b, the genetic distances between samples from the continental regions were based on the respective sharing of their own set of continental haplotypes^12^. In this case, the closest genetic affinities between samples from regions within continents separated the Northern African regions far from European regions along the X coordinate axis. Again, the eastern and western islands were separated along the Y-axis although now Gran Canaria showed the greatest genetic affinity to northern Africa while the western islands showed a closer genetic relationship to the European regions. A sign test based on the number of haplotypes shared between groups and those unique to each group showed that the groups statistically differ (*p* = 0.001). However, due to the high haplotype diversity of the total indigenous sample, it is uncertain whether the eastern and western islands samples originated from different populations. In the following analysis on haplotype differences between the two groups of islands, it was assumed of northern African provenance all the haplotypes with matches in North Africa although they were also present in other regions, and of European provenance those haplotypes with exclusive matches in Europe. Prominent or exclusive haplogroups in the eastern islands included H1 (16239), H1ao (16278), H3r (16126), H4a1e (16362), T2c1d3, U5, U6a, U6c, M1, and L3b1a12 (Table S1). U6a, U6c and M1 have a pan-Mediterranean range and U6a and M1 have been in Northwest Africa since the Pleistocene^24^, which also implies H1 (16239) and H3r (16126)^25^. A recent study has extended the geographic range of H4a1e to southern Egypt prior to Roman and Greek influx^26^. In addition, some T sequences have localized matches: T1a (16126-16154-16163-16186-16189-16294) in Algeria^27^, T2c1d3 (16092-16126-16292-16294) in Morocco^28^ or T2c1d3 (126-292-294-362) in the Near East. However, basal U5b1 haplotypes are present in a broad geographic range from the Western Sahara^29^ and Mauritania^30^ to Mediterranean Africa (Table S1). On the opposite side were the haplotypes of haplogroup L3b1a12, whose African location of origin remains unknown^6^. In relation to haplotypes having probable European origin, H1e1a9 (13934) and HV (16316) stand out for their exclusive matches in Italy. Nevertheless it has to be mentioned that an ancestral type of H1e1a was detected in Chalcolithic–Middle Bronze Age samples from Portugal^31^. In the western group, northern African heritage was represented by several haplotypes derived from the H1 haplogroup (Table S1). The H1cf complete mtDNA haplotype had its closest relative in Algeria^9^. The majority of J haplotypes in the indigenous population were from the western islands, and the J2a2d1 branch seemed to originate from northwest Africa, and was present on all western islands except El Hierro (Table S1). Haplotypes of haplogroup U6b1a primarily showed the greatest northern African contribution to the western islands, having highest incidence in La Gomera^10^ and being absent from El Hierro. Although not detected on the African continent, U6b1a has its closest sister clade (U6b1b) in Morocco^19^. The U6b1a haplotypes trace to South America and the Iberian Peninsula after the forced migration of indigenous canarian people after the conquest (Table S1). The high incidence of haplogroup H haplotypes indicates primary European contributions (Table S1). For example, the H1 (16292) haplotype was detected in all the western islands except La Gomera, with matches in the Iberian Peninsula and Italy. For the J haplotypes other than J2a2d1, J1c3 and J1c2c2 present in Tenerife had exact matches in the Iberian Peninsula and in Italy and France, respectively (Table S1). La Gomera had an enigmatic N1b1a7 lineage with an exact match in the Middle East alone^32^. La Palma also harbored two haplotypes of macrohaplogroup N. W1e1 had matches in the current populations of the Iberian Peninsula and Italy, being detected since the Neolithic in Catalonia^33^, indicating its ancient Iberian Peninsula presence. The other, a specific derivative of X3a (16111-16189-16223-16278), also had a unique match in the Iberian Peninsula (Table S1). Finally, El Hierro had a rare U5a1b4 haplotype solely found in France and a rare U7 haplotype (16309-16318T) whose nearest matches were in the Iberian Peninsula and Italy, but that also is in Egypt^34^.

Finally, around 12% of the original indigenous lineages traced to sub-Saharan Africa, although some also were found in northern Africa, and approximately 30% had exclusive matches to regions where the Portuguese slave trade peaked (Table S1). This resembles the current populations of the Macaronesia Islands of Madeira and the Canarian archipelago, where about 40% of their sub-Saharan L sequences have exact matches in Cape Verde and Sao Tomé and Principe, which were main outposts of the Portuguese Atlantic slave trade^17^.

### New coalescence ages for the Indigenous lineages

Some of the first radiocarbon dates placed the indigenous settlement of the Canary Islands back to late Neolithic times, which agreed with their cultural level^35^ and with the first coalescent age estimations obtained for the Canary islands mtDNA autochthonous lineages U6b1a and U6c1a around 5000 ya^36,37^. However, those old radiocarbon dates have recently been reconsidered due to the inappropriate material used. New and revised archaeological dates and demographic inferences have concluded that, a permanent settlement on the islands prior to the first millennium AD is highly improbable^3^. In parallel, studies of the mtDNA evolutionary rate^15,38^ have found that it is dependent on the population size and that a rate of one mutation every 3624 years extensively used in human phylogenetic analysis^13^ is inappropriate to apply to relatively recent events. For shallow phylogenetic trees that concur with the time frame studied here, an alternative evolutionary rate of one mutation every 1400 years was proposed^14^ which have been used in the present study. Notably, this predicted fast evolutionary rate for recent times has been empirically corroborated recently by an extended pedigree analysis, using the entire mtDNA genome, obtaining a mutation rate of 5.8 × 10^−8^ (95% CI 3.10–10.8 × 10^–8^) mutation/site/year that nicely overlaps with the one used here of 4.33 × 10^–8^ (95% CI 3.90–4.82 × 10^–8^) mutation/site/year^39^. Applying this evolutionary rate to the phylogenetic trees (Figs. S1 to S6) of the 16 indigenous lineages that are supported by complete mtDNA sequences, for the indigenous^6,20^ and current populations^12^ of the Canary Islands (Table S4), the coalescence ages ranged from 2,333 (95% CI 2300–2368) ya for the H1cf (16260) clade to 382 (95% CI 361–401) ya for the Gran Canaria autochthonous lineage L3b1a (@16124). It deserves mentioning that H1cf and H1e1a, the oldest lineages, both belonged to the European haplogroup H1. For the former, the closest sequence to the Canary cluster was an Algerian sequence^9^ and for the latter an Italian sequence (Table S1). These haplogroups were followed in age by J2a2d1a and U6b1a with main introductions in the western Islands, and U6c1 limited to the eastern islands, whose ages located them in the Canarian archipelago between the second and the fifth centuries AD. At first, this apparent continuous range of ages could be compatible with a permanent flux of migrants to the Archipelago. However, this contrasts with the important genetic drift effects observed in the islands of La Gomera^10^ and El Hierro^23^ and the relatively high genetic differentiation found between the main islands of Tenerife and Gran Canaria^6^. These results are more in line with successive but discrete migrations that did not affect all of the islands equally. Thus, taking into account the relative proximity of their respective ages, we subdivided the indigenous lineages into three discrete time intervals (Table 4). The oldest group comprises the five lineages (H1cf, H1e1a, J2a2d1, U6b1a, and U6c1) commented above. Lineages of the middle aged group (W1e1, X3a, and U5a1b4) may have arrived to the Archipelago at the beginning of the twelfth century affecting only the western islands, coinciding in time with internal population growth marked by the autochthonous U6b1a1 lineage. These three lineages would have had a European origin instead of Arab. The third and most recent group coincides with the period of the European colonization of the Archipelago (from 1402 to 1496 years). In it U6a* represents a set of current Canarian sequences belonging to subgroups U6a1a1 (16239), U6a3a1, and U6a7a1b, all also detected in the indigenous sample (Table S1). These three clades had Chalcolithic expansions in Europe^18^. From them, it is particularly interesting the case of U6a7a1b that is related to the Sephardic radiation and historical diffusions to the American continent^18^. Clades H4a1, T2c1d3 and T2c1d1c could signal the post-conquest Moorish slave trade^6^, while the L sub-Saharan African members seemed to result from the Atlantic slave trade practiced by Portuguese traffickers^12^. Predictably, age differences between groups 1 and 2 (*p* = 0.0007) and between group 2 and 3 (*p* = 0.0026) were highly significant.

## Discussion

### Contamination problems in ancient DNA studies

Due to the availability of many human mtDNA sequences in data banks, for which the recent contribution of Canarian samples is remarkable^12^, rare or incomplete indigenous haplotypes published in earlier studies on ancient DNA from the Canary Islands^8^ appear related to lineages sampled in the current population, highlighting their potential authenticity. Paradigmatic are the cases of H* (16290) in La Palma, J1c2e2 (16069-16126-16278-16366) in Tenerife, L3d1b3a (16124-16223-16256-16311) in La Gomera, or U5a1b4 (16093-16192-16256-16270-16362) in El Hierro^12^. Remarkable are also other indigenous types detected in South America regions with demographic ties in the Canary Islands (Table 2), and those identified in continental areas where their potential ancestors originated (Table 3).

**Table 2:** Indigenous mtDNA haplotypes present in the historic or current Canarian population but absent in North Africa.

Haplotype	Haplogroup	IP	FR	IT	Other
126	H3r	14	9	35	
239	H1	14	9	10	
292	H1	8	9		
316	H1bw	13		10	
192 260	H3v				Chile (67)
069 126 278 366	J1c2e2	8	9		
163 172 219 311	U6b1a	43			Pto.Rico (66)
048 163 172 219 311	U6b1a				Uruguay (65)
092 163 172 219 311	U6b1a				Chile (67)
223 292 295	W1e1	35		10	
223 278 311 362	L3b1a12				Dominican (44)

**Table 3:** Indigenous mtDNA haplotypes absent in the historic and/or current Canarian population.

Haplotype	Haplogroup	NW	NA	IP	FR	IT	Other
067	HV1	11	12	37		10	
086	H1	6	20	14	9	10	
213	H/HV/U/R	21	12	45	48	26	
223	H1		19	29	9	24	
265	HV/R	16		14	9	24	
302*	H1				9	19	
172 278	H1	16		51			
145 213	H/HV/U/R						
260 278	H1cf						Yemen (62)
129 294*	H/HV/U/R				9		Frisian (63)
316	HV						Iran (38)
189 316*	HV					73	Iran (38)
069*	J1c3			13		19	
126 294	T2e	11	19	37	9	24	
126 224 292 294	T2c1d3						Romania (58)
126 255 292 294	T2c1d3						
126 292 294 362	T2c1d3						Iraq (57)
126 154 163 186 189 294	T1a		53				
270 294*	*U5a2a*			41			
239 278	*U6a1a1*						
172 219 221 224 278	U6a						
169 172 189	U6c1	6	34	45	44		
129 169 172 189 213	U6c1						
309 318T*	U7			29		24	
145 176G 223 297 311	N1b1a7						Armenia (63)
111 189 223 278*	X3a			13			
126 223 262 320	L3e2b						
223 278 390	L2	6	19				
223 239 278 292	L2e						
111A 145 184 223 239 278 292 311 355 390 399 400	L2e						Senegal (6)

The absence of matches with any published mtDNA sequences of some indigenous haplotypes might be due to insufficient sampling in their putative areas of origin but, in some cases, as evidenced here, may indicate contamination, mixed up types or incomplete sequencing, which has led to the identification of the most probable indigenous haplotype and its contaminant (Table 1). Finally, some indigenous haplotypes, with potential relatives in Europe that are not detected in historical or present-day Canarian populations, may represent pre-conquest male limited incursions that did not transmit this maternal marker. Other empirical data appear to support this hypothesis. The Y-chromosome haplogroup I-M170 is a predominant European male-lineage. It has a frequency around 9.8% in the Iberian Peninsula, but is rare in northern Africa (0.002%), a difference that is statistically highly significant (P < 0.0001). Curiously, haplogroup I-M170 reached a frequency of 6.7% in a Canarian indigenous sample^40^ that also significantly differs from northern Africa (p = 0.0097). These results could indicate a male-mediated European gene-flow on the indigenous population before the Spanish Conquest or, alternatively, a strong contamination/admixture of the indigenous remains with potential European remains. Although more recent techniques of enrichment and sequencing of ancient DNA make it easier to identify contamination, the reassessment of doubtful sequences with the panel of publicly available sequences as performed in this study will continue to be a useful strategy.

### Lack of date and context of archaeological samples

Donated archaeological samples should be accurately dated and contextualized following precise radiocarbon hygiene protocols. Regrettably, this was not the case in the first ancient DNA studies on Canarian indigenous material, in which the samples consisted of non-individualized, loose teeth, theoretically obtained from indigenous sites roughly dated around 1000 ya. Thus, in order not to duplicate samples, geneticists opted to use a single tooth type, preferably the left canine^8^ for all DNA extractions. Although molecular results from that material yielded important information, including the presence in the indigenous sample of several predicted founder lineages as U6b1a^22^, the critical re-analysis performed here suggests that those putative indigenous samples contained a jumble of samples that, in addition to indigenous ones, included European remains from the conquest period, remains of Moorish and sub-Saharan Africans brought to the islands by the Europeans as forced labor and, probably, remains of fugitive Sephardic people. Thus, the supposedly high genetic diversity found in the Indigenous sample^8^ was in part the result of heavy archaeological contamination. This seems to be confirmed by more recent molecular studies on dated and contextualized archaeological material, for which observed genetic diversity is appreciably lower^6,7^. Another effect of the absence of precise archaeological dating is that the long-debated hypotheses of one or more colonization waves to the Canary Islands depends on the coalescent age of those indigenous lineages that remain represented in the current population^12^.

### Molecular age for a permanent indigenous settlement

The mtDNA evolutionary rate of humans may have accelerated in recent times^14^. Applying this faster rate to calculate the coalescent ages for those indigenous lineages that remain represented today (Table S4), revealed molecular ages between 2300 and 2185 years ago for the two oldest lineages, H1cf and H1e1a (Table S4), that is, two or three centuries BC. These molecular ages are earlier than the recent archaeological estimates, dating the first settlement of the Canary Islands to two or three centuries AD^41^, but are much closer to each other than those previously proposed^13^. On the other hand, age differences among lineages, and their heterogeneous settlements on the islands, provides clues to address questions such as whether the Archipelago was colonized during one or several immigration waves, or whether the pre-conquest settlers arose from one or more genetically heterogeneous populations. Focusing first on the oldest group (Table 4), two lineages (H1e1e and U6c1) showed a wide Mediterranean geographic range, including Italy and northern Africa, who exclusively settled on the eastern-Canary islands. On the other side, three lineages (H1cf, J2a2d1, and U6b1a) showed a prominent or exclusive trace to the western islands, of which at least two (H1cf and U6b1a) appeared restricted to northwestern Africa. As the range of their ages did not allow us to significantly separate these lineages, alternative possibilities may involve only a single heterogeneous wave, or coetaneous heterogeneous waves, of settlers who colonized different groups of islands. This contradicts an earlier suggestion that the H1e1a, H4a1e, L3b1a, and U6c1 clades had an asymmetrical implantation in the eastern islands that may signal a late secondary settlement on these islands^6^. It further was inferred that most sites where these lineages were sampled had radiocarbon dates around the thirteenth century. However, the late age of the sites sampled does not guarantee that those lineages did not settle on the islands earlier, as suggested by their coalescence ages (Table S4). The second group of lineages indeed could point to the existence of a second wave of colonizers affecting the western islands in an interval from the end of the tenth to the beginning of the twelfth centuries albeit, if it occurred, it had a minor impact on the maternal genetic pool of the islands population. However, the incorporation of those maternal lineages, into the western islands may have been due to early pre-conquest European sporadic landings. The third group is a set of lineages that likely became incorporated into the Canarian population during the European colonization period. As previously mentioned, the clades T2c1d3 and T2c1d1c although not detected in indigenous remains suggested an autochthonous radiation, which could indicate the post-conquest forced Moorish incorporation in the eastern islands^6^, while the sub-Saharan African L haplotypes during the same period could have resulted from the Portuguese Atlantic slave trade^12^. Note, however, that the eastern islands L3b1a lineage likely should be excluded from this post-conquest input as it was detected in individualized remnants radiocarbon-dated to 1,116 + 26 years BP^20^. Because of this, the shallow age of coalescence obtained for the clade (Table S4) may be attributed to a possible loss of some divergent haplotypes due to genetic drift. Future knowledge of the place from where the L3b1a and U6b1a lineages came to the islands will help to resolve the precise origin of the indigenous Canarian settlers. Finally, since the lineages of the second and third groups mainly belonged to the western islands, their relative genetic closeness to those from European regions (Fig. 3b) likely is not due to differentiation between indigenous populations but due to contamination of the archaeological samples.

**Table 4:** Settlements on the Canary Islands based on coalescence age and phylogeography of Indigenous mtDNA lineages.

Lineages	Period	Mean age	95% CI	Islands’ group	Origin
H1cf				Western	Algeria
H1e1a				Eastern	Italy
J2a2d1	124 AD	1.876	1426–2325	Western	Tunisia
U6b1a				Western	Morocco
U6c1				Eastern	Italy, Morocco
W1e1				Western	Italy
X3a	1117 AD	883	809–956	Western	North Africa
U5a1b4				Western	France
*U6b1a1*				Canarian	Autochthonous
U6a*				Eastern	North Africa
H4a1				Eastern	North Africa
L2e				Western	Senegal
T2c1d3	1430 AD	570	435–704	Eastern	North Africa
L3d1b3a				Western	Morocco
*T2c1d1c*				Eastern	Autochthonous
L3b1a				Eastern	Unknown

With the available ancient mtDNA data, it could not be discerned whether more than one wave of pre-conquest colonizers occurred as some archaeological investigations suggested^42^, but it does seem that a genetically heterogeneous population or populations likely colonized the Canary Islands in an asymmetric way around the first millennium AD. Earlier studies about physical anthropology of the Canary Islands indigenous people already pointed to the existence of a physically heterogeneous population. In one of those, a clear sub-Saharan African component was detected^43^ although it was ruled out after the analyses of dermatoglyphics and haptoglobin types in the extant population, which did not reveal any sub-Saharan African affinities. To explain the discrepancy, it was suggested that some sub-Saharan African skulls, from the post-conquest slave trade, could have been included in the analysis inadvertently^44^. However, in this regard, it should be noted that, due to genetic recombination, a sub-Saharan African immigrant genome would have been diluted into the recipient population in a few generations, whereas a mtDNA lineage would retain its African roots without modification. More thorough analyses concluded that the skulls of the first islanders might be explained as mixtures, in varying proportions, of two ancestral types: the robust Cromagnoid from northwestern Africa and the gracile Mediterranean Capsian^45^. Both types were present in the main islands of Tenerife and Gran Canaria, with the Crogmanoid features being more prominent in the northern and mountainous regions and the Mediterranean along the coasts; in addition, the Crogmanoid type was best preserved in La Gomera^45^. However, on the contrary, a more recent study based on dental morphological measures for the same indigenous populations of La Gomera, Gran Canaria, and Tenerife found that inter-island dental differentiation was so minor that it did not require any hypothesis of separate founding populations^46^. The accumulated biological data on the first islanders is still far from forming a coherent body, and their coupling with the archaeological data only reaches some specific agreements, such as that their ancestors came from northern Africa and that a permanent settlement on the islands cannot go back much further than the beginning of the first millennium AD. Nevertheless, the ancient mtDNA information reanalyzed here is already enough to support some of the several hypotheses formulated to explain where the first settlers originated, how they arrived at the archipelago, and how they settled on the different islands.

### In support of a Roman-mediated indigenous settlement of the Canary Islands

**Figure 4:**
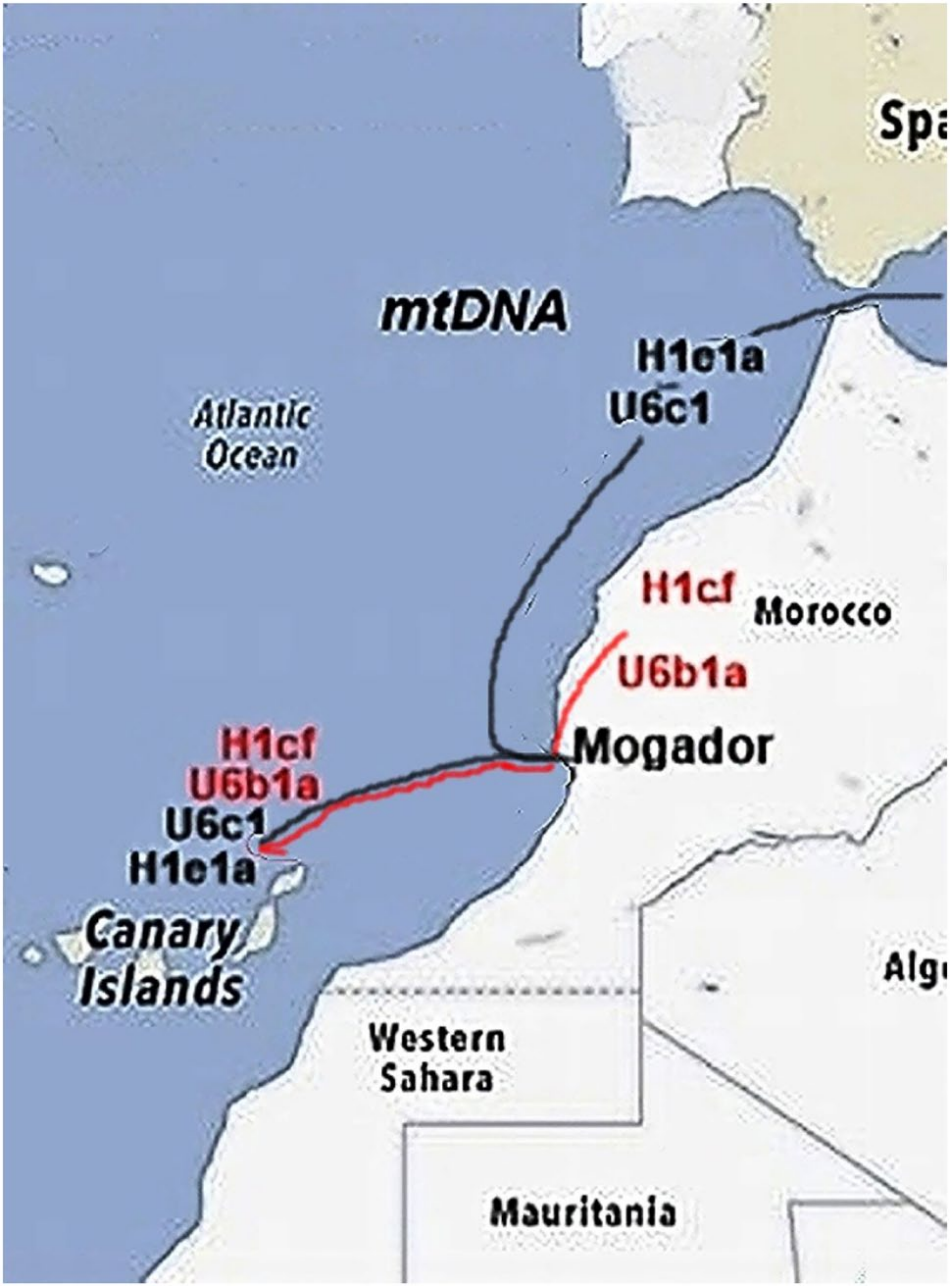
Putative routes followed by the indigenous carriers of the northern and northwestern African haplotypes to the Canary Islands.

The first question about the indigenous Canarian population that seems to be resolved is when they arrived on the islands since both the archaeological and genetic data place it around the first millennium AD, questioning previous hypotheses proposing Neolithic or Phoenician-Punic settlements^47^. The genetic support for settlement in Roman times is the lack of indigenous lineages in the indigenous^6^, historical^48,49^, and current Canarian population^12^ with coalescent ages older than this epoch. However, earlier arrivals to the islands that did not leave a genetic trace cannot be ruled out. Indeed, there is archaeological evidence that Romanized people landed on the eastern islands and established a purple dye extraction workshop on the islet of Lobos^50^. The high economic benefit that the purple trade achieved in Roma provides additional support explaining the far-flung and costly maritime voyages. However, for this business to be profitable, a small workshop like the one discovered on the islet of Lobos would not yield enough production. *Stramonita haemastoma,* the mollusk from which the purple dye was extracted in Lobos, is also abundant and easy to collect on some coasts of the other Canary Islands^51^. Thus, although the main exploitation centers must have been in the eastern Islands, where the frequency of the mtDNA Mediterranean lineages was greater, it seems likely that other purple workshops, still not detected, were established along the Archipelago at the same time. Furthermore, due to depletion of the raw material, migration among islands likely became common. However, except for a few potential Latino-Roman rock scripts on the eastern islands, there is no trace of Roman culture in the Canarian pre-Hispanic archaeology^52^. Because the coalescent ages (Table 4) of mtDNA haplotypes from concentrated ancestry in Northwest Africa (H1cf and U6b1a) are similar to those in the Mediterranean range (H1e1a and U6c1), they might have coexisted on the islands with little cultural or genetic exchange, which raises the possibility of independent arrivals for each group at the same time. However, from the beginning of the conquest, written records indicated the native islanders, although good swimmers appeared to lack navigation skills and there was no communication among islands^53^. This led to the widespread idea that they might have been voluntarily or involuntarily transported to the islands by people with the maritime capacity to do so^54^. In favor of the first option is the fact that these island settlers brought livestock and seeds with them for their future subsistence, implying that it was a programmed migration, which presupposes previous knowledge of destination. But if this was the case, why did they not bring with them other technological advances already in use in northern Africa at that time? This includes bronze or iron tools and weapons, the Roman plow, and the ceramic lathe, just to mention a few. The second option, that they were forced to migrate, resolves these questions and could explain the genetic heterogeneity of the indigenous population. The exploitation of purple was a hierarchical business. At the top were the elite, which had the economic and technological power to carry out this undertaking. Following were the artisans specialized in dyeing the fabrics, then the workforce capable of extracting the dye, and, finally, the slaves that had to collect the mollusk; both of the latter likely were brought to the Canary Islands. Most likely, the dye extractors were recruited from the already settled Mediterranean purple dye workshops, while the slaves, for economic reasons, would have been captured or bought in the vicinity of the Archipelago in places such as the Atlantic Moroccan port of Mogador (Fig. 4).

When the purple dye industry ceased being profitable, those people likely were left to fend for themselves on the islands. For subsistence reasons, goats and barley accompanied people on their previous inter-island transfers, making their subsequent adaptation possible. Notably, the fact that the indigenous barley has been continuously cultivated since the pre-Hispanic colonization of the islands^55^ and the persistence of indigenous goat breeds^56^ suggest that there were no major intrusions into the islands until their European conquest.

## Methods

### Samples

Partial and complete mtDNA sequences of the indigenous^6,8–10,20,23^, historical^48,49^, and present-day Canary Islands population samples^12,17,22^ were compiled from prior published studies (Table S1 and supplementary bibliography). To find the closest matches to the indigenous Canarian sequences, nucleotide rare variants and the co-occurrence among point variants were used to search within known haplogroups, and short sequences, including total or partial haplotypes, were used to query the whole dataset in the following databases: NCBI GenBank (http://www.ncbi.nlm.nih.gov/genbank/, Mitomap (http://www.mitomap.org/MITOMAP^57^, Ian Logan 2020 (http://www.ianlogan.co.uk/sequences_by_group/haplogroup_select.htm, Empop database (http://www.empop.online/haplotypes^58^, and AmtDB (http://www.amtdb.org). Mutations that have not been previously found in any haplotype of a given haplogroup were considered putative contaminant mutations and further analyzed on other haplogroup contexts. Rare mutations that appeared on different haplogroup backgrounds were considered phantom mutations. In total 336 mtDNA indigenous sequences were reanalyzed of which 288 were HVSI partial sequences (16,000 to 16,400 range) and 48 were complete mitogenomes. In addition, 3246 northern African and 10,960 European sequences were screened in search of haplotype matches. Detailed sample sizes for each island and continental regions are specified in Table S1.

### Sequence classification

Sequence assignment to the corresponding haplogroup and its sub-haplogroups was checked using HaploGrep version 2, https://haplogrep.i-med.ac.at^59^, and PhyloTree build 17 version, http://www.phylotree.org^60^. Sequence variants were scored with respect to rCRS^61^. The output raw trees were manually checked and refined. The hotspot 16,519 mutation and indels around nucleotides 309, 522, 573, and 16,193 were excluded from the trees and from the statistical analysis. Partial sequences that could not been unambiguously classified within specific haplogroups were discarded in all analyses.

### Population based statistical analyses

Due to strong genetic drift effects observed in the Canary Islands indigenous populations^6^ and to compensate for the dominant influence of the most common haplotypes in the frequency-based pairwise distances, a match-based distance method proposed elsewhere was used^12^. For statistical haplotype comparison of the western (Tenerife, La Gomera, La Palma, and El Hierro) and eastern (Lanzarote, Fuerteventura and Gran Canaria) population samples from the Canary Islands, we used a Hamming distance in which positive matches (1) were compared against negative matches (0), applying a sign test for categorical data (https://www.graphpad.com/quickcalcs/. Haplotype overlap among the northern African and the Mediterranean regions of Europe studied were graphically represented by Venn diagrams. A binary matrix indicating presence (1) or absence (0) of the indigenous haplotypes on each island and the continental regions sampled was the input for these analyses (Table S2). From a pairwise match-based distance matrix (Table S3), principal coordinates analysis was performed as implemented in the GenAIEx 6.51 web site^62^. Fisher’s contingency tests and t-tests were calculated using the graphpad calculator (https://www.graphpad.com/quickcalcs/.

### Coalescence age estimations

The coalescence ages for the putative autochthonous Canarian lineages were calculated using rho statistics^63^ and a revised substitution rate of one mutation every 1400 years (95% CI 1261–1539) based on the most recent period of human demographic history^14^. Seqbot (https://evolution.genetics.washington.edu/phylip/doc/seqboot.html) was used to generate 3600 bootstrapped mtDNA alignments to calculate the rho statistical error for each autochthonous founder lineage using the python-based script *‘bootstrap rho.py’* available at (https://github.com/genomicsITER/mitogenomes/tree/main/CanarymtDNA^12^.

### Institutional review board statement

This study underwent formal review and was approved by the Ethics Committee for Human Research at the University of La Laguna as proposal NR157.

### Methods statement

All methods were carried out in accordance with relevant guidelines and regulations.

### Supplementary Information

Supplementary Figures.

Supplementary Information.

Supplementary Tables.

## Data Availability

The data present in this study are available in the article and Supplementary Materials.
